# Considerations Practiques Sur Les Neuralgies De La Face, i. e. Practical Observations on Neuralgia of the Face

**Published:** 1833-10-01

**Authors:** 


### Considérations Pratiques sur les Neuralgies de la Face, par M. *Halliday*, Docteur en Medicine, des Facultés d'Edimbourg et de Paris. 8vo. pp. 170.

It is only within the last fifty or sixty years that the attention of medical men has been properly directed to the study of those most painful affections of the nerves, known under the title of neuralgia, tic douloureux, trismus dolorificus, &c. We find, indeed, allusions to them in some of the older authors; but these are indistinct and imperfect; and it was not until André, of Versailles, and Dr. Fothergill, of London, about the middle of last century, wrote their interesting papers, that the disease was recognized as a legitimately idiopathic and independent member of the nosological catalogue.

It had been confounded with rheumatism, gout, cephalalgia, toothache, &c.; physicians seeming to have forgotten, or never to have been rightly aware that the nerves, like all other parts of the body, may be originally involved in morbid action, as well as suffer sympathetically.

In the consideration of neuralgia, it is of first-rate importance to keep this remark steadily in view; the accuracy of our prognosis must, in a great measure, rest upon it, and the success of our treatment be thence materially affected. As the main value of Dr. Halliday's monograph consists in the valuable cases which he has assiduously collected together and arranged, we shall present our readers with some of the most interesting and instructive of these, and then cursorily glance over the second part, which is devoted especially to the detail of the various proposals which have been made for the relief of this truly dreadful, and sometimes incurable affliction.

*Case* 1. A merchant at Rouen, 65 years of age, had suffered more or less from excruciating darting pains in the eye-ball, cheek, nose and gums, for eight or nine years. At first they lasted only for a moment; a sudden flash of agony, and then over. They were more frequently felt in the evening than at other times. They gradually became longer, and returned more often: and for three successive months in one year, the patient had little respite. Most of his teeth had been extracted, but all to no purpose. Subsequently to this dreadful attack, the paroxysms had become more severe, and frequently lasted for eight days at a time. In Summer he suffered much less than in Winter. The disease gradually increased in violence, and left the poor sufferer little respite; for days together he had sometimes not two hours perfect freedom; and the horrid stabs which at first lasted only for a second or two, continued now for several minutes. It seemed as if all the nerves of the eye-ball were torn asunder; the pain sometimes darted from the eye-brow to the top of the head; but the seat of the pain was not always in one part; but was in this twig one day, and in that another. A great variety of remedies were used, but all in vain. He then applied to a physician at Rouen, who recommended the use of the magnet. He immediately experienced relief from its application. The pain was assuaged as by a charm, and left only a feeling of numbness in the part. The patient carried the magnet constantly with him: it was of such a power as to raise a weight of six pounds. During a full twelvemonth the patient had comparatively great comfort; but after that his enemy threatened to return with its former violence; the paroxysms were, however, always mitigated but not subdued by the magnet. The history of this case ceases abruptly here.

The branch of the ophthalmic nerve, which is the most, or at least as frequently affected as any other, is the frontal, or supra-orbital. The external twig, which supplies the forehead, generally suffers; sometimes, however, although rarely, it is the internal, or that which is dispersed on the inner canthus, frontal sinuses, and lacrymal gland. The following is an example of it.

*Case* 2. A female, aged 33, while affected with catarrh, exposed herself to a current of cold air. In the course of a few hours she experienced an intense pain over the left frontal sinus, darting and shooting through the orbit with electric violence; the eye throbbed vehemently, and the tears which flowed were burning to the cheek. The paroxysm lasted for several hours, and then gradually abating, left her entirely free until the following morning at the same hour.

Emollients, opiates, and local bleeding, were of no use; and the patient of her own accord took some very strong coffee, as she had heard of its utility against migraine. The attack upon that day was less severe; and by continuing the coffee for some time she gradually recovered. She remained free of her nervous enemy for four years; it then returned after much mental distress. Again she had recourse to her coffee, and derived equal benefit as before. On the following year, in the cold month of November, it came back once more; and now, unfortunately, resisted the use of coffee, and also of opium, bark, and many other medicines. Slight relief was experienced by drinking copiously of warm diluents.

A similar case is reported by Dr. Fothergill: it occurred in an elderly lady, whose health in other respects was good. Hemlock gave her more relief than any other anodyne. She continued to suffer from her tormentor, to the day of her death. Van Swieten has related, in his Commentaries, several good cases of frontal neuralgia; he knew the efficacy of the cinchona bark, when the paroxysm was periodical.

The nasal twig of the ophthalmic is occasionally the seat of the pain.

Meglin, in a very excellent memoir, published in 1816, at Strasbourg, gives a case in illustration. A peasant, after a fit of violent passion, was seized with an intense pain in the right upper eye-lid, darting thence to the nose. It came on regularly about seven o'clock in the morning and lasted until noon, when it ceased, but only to return on the following morning about the same hour. The cinchona was given for three or four days; but it quite failed, and Dr. M. prescribed the following pills, which effected a perfect cure.

℞. Extracti hyosciami,
——— Radicis valerianæ,
Oxydi zinci sublimati, āā. ℈ii.

In pil. mediæ magnitud. dividend. The dose to be gradually increased from two or three to ten or twelve, morning and evening.

When neuralgia affects the dental twig of the superior maxillary nerve, the patient, as well as his medical attendant, is apt to suppose that the pain proceeds from a diseased tooth, and although it may be sound to all appearance, it is extracted, with no relief to the suffering. This error has been, and still is, so repeatedly committed, that we earnestly advise our readers to consider the following cases, which are published by Dr. Halliday. In 1793, a farmer was annoyed with toothache on the right side of the upper jaw; the teeth were sound, but the gums were redder than natural, and the cheek somewhat swollen. A dentist, who was consulted, upon finding that one of the back teeth, though sound, was tender when touched, recommended its extraction, as he thought it probable that the fangs might be diseased. The patient submitted, but the pain raged as much as ever. The dentist proposed to cauterize the socket; this was done, and the agony increased. By soothing topical remedies, partial relief was obtained for a time, and again the horrid sufferings returned. His life was so wretched, that in the agony of his pain he committed suicide. The particulars of the preceding case will be found in a brochure published by that intelligent dentist, M. Duval, of Paris. A similar and equally severe case occurred in the person of a lady, who at the time she consulted M. Meglin, had had nine of her teeth successively extracted; after each operation, except the last, the pain had ceased immediately, but returned at different times. She had moreover tried a great number of remedies, but without avail. M. M. ordered the pills of the extracts of hyosciamus and wild valerian, and of the white oxide of zinc. In the course of three weeks she was cured, and has had no relapse.

Professor Leydig, of Heidelberg has written an interesting report of a very severe case of infra-orbital neuralgia. In 1793, a musician, whose health had previously been very good, was seized with an intense pain, beginning in the occiput and extending round the head to the frontal region. So violent was it that the patient was forced to throw himself down on the bed, and remain quite motionless till it passed over. The intervals between the paroxysms were at first nearly three weeks; but gradually this period contracted, and the fits seized him every day, and sometimes twice in 24 hours. For several years he was a martyr to these cruel sufferings; all means, including cinchona, that were tried, had failed. He caught an ague, and bark was freely given; the ague was cured and so was the neuralgia, at least for a year or two, for then it once more returned: the act of sneezing, blowing the nose, speaking, chewing, shaving, &c. brought it on; and the stabbing and tearing pains were accompanied with convulsive twitches of the muscles of the face, and with weeping of hot tears from the eyes. Towards the middle of 1800, the patient was more afflicted than ever; for now the paroxysm came on spontaneously, and seemed to be independent of any movements of the mouth or jaw. The pain commenced about the root of the right upper canine tooth, which during the paroxysm was so excessively sensitive, that the gentlest touch even of the tongue against it, caused an insufferable pang, which shot like lightning through the lids and globe of the eye, the right side of the nose, and cheek; the muscles of these parts were convulsively twitched, and the mouth drawn to the affected side. Each paroxysm usually terminated by a copious flow of mucus from the nostrils. An experienced physician tried the effects of almost every medicine in the Materia Medica; many seemed to afford relief for a time; but the respite thus obtained was short and delusive. One year he took the waters of Wisbad for eight weeks; and certainly derived benefit from their use; on the next year they quite failed. In 1804, his sufferings were, if possible, still more agonizing; for the pain now shot up along the forehead to the hairy scalp; the eye was rolled outwards, and the poor patient described the torment as if hooks had been plunged into the eye, and they were dragging it from the socket. The left side of the face, which hitherto had been exempt, was now affected with convulsive movements, and the sense of hearing had become much obscured. The slightest touch of the nose, cheek, ear, or right side of the neck, the odour of vinegar, or any volatile aromatic, the least breath of wind, the vibration caused by musical instruments, would frequently induce a paroxysm, or add much to its severity, if it were on.*

Some days the patient escaped altogether; while, on others, he had not fewer than a hundred paroxysms in 24 hours. In cold, clear weather, he was much better than when it was warm and damp. Each paroxysm lasted usually for about a minute, and terminated by a flow of tears and by a discharge from the nostrils; if this did not take place, the paroxysm was immediately repeated. Carrying on the history, we are told that, in 1805, the patient suffered even yet more dreadfully than before; the pain now extended to the palate, velum, and back of the throat; for five months he had not one hour of calm sleep. His general health had suffered greatly, not only in consequence of the acuteness of suffering, but also from the use of immense doses of opium, which he was in the constant habit of taking.

Leydig advised him to submit to the division of the infra-orbital nerve; and the operation was performed on the 22d of June. The nerve was freely and fairly cut across, and the knife drawn firmly along the bone, in order that no filaments might escape. For several days after, the patient experienced a few atacks of pain; but these were now infinitely less severe, and the ultimate result was most gratifying.

At the date of the report, 13 months after the operation, the patient remained well and free from any return; only during Spring and Autumn, (which were the seasons when he used to suffer most severely) the right side

* There thus appears to be a sort of resemblance between the phenomena in violent neuralgia and some of those of hydrophobia; the horribly exquisite sensitiveness of the skin to the gentlest impressions is a striking feature of both.

of the face was exceedingly sensitive to any impression. The painless, convulsive twitches of the left side of the face, and the anæsthesia of the upper lip and wing of the nose, on the other side, still continued.

Hitherto, our attention has been occupied with the nerves of the first and second divisions of the fifth pair, and now we arrive at the third, or inferior maxillary nerve. The twig most frequently affected is the mental; sometimes, however, it is the gustatory, and, at other times the small branches distributed to the masseter, pterygoid, &c. muscles. Such cases are not uncommonly confounded with neuralgia of the portio dura.

An old man, for eight years before his death, suffered from paroxysms of dreadful pain in the right side of the tongue; these were often brought on by the slightest movement of it, as in speaking, chewing. The pain was so agonizing, that the face was distorted with convulsions. The oxyde of zinc, taken internally, and also sprinkled on the tongue, afforded him some relief. In two similar cases, reported by André, cures were effected by cauterization.

### Neuralgia of the Portio Dura.

It is a mistake which has been long, and still is, committed by many good authors, to suppose that the facial nerve is the most frequent seat of tic douloureux. MM. Boyer and Delpech first pointed out the inaccuracy of this doctrine; but, even according to them, it is more frequently affected than present experience warrants us in believing. Simple and primitive neuralgia of the portio dura is very rare. Dr. Halliday has not been able to find more than four or five examples of it, among the numerous authors he has consulted. M. Ribes, in the second volume of Majendie's Journal of Physiology, has narrated one. The pains were most excruciating, and, in the course of a short time, they began to affect the organs of digestion, and induce vomiting, so that nothing could be retained on the stomach. The patient was cured by quinine. In another case, which also occurred in a female after severe mental distress, benefit was derived from the exhibition of belladonna, and the hyperoxymuriate of potass. In a patient of Pelletan, at the hospital of the School of Surgery, the disease seemed to have been caused by the injury of some small twig, during arteriotomy at the part. The cautery was used, but failed, and the patient left the hospital.

Mr. Waton, in the Journal of Medicine and Surgery for 1793, has reported a very instructive example. A young military officer, of a healthy constitution, had suffered for a year and a half from repeated attacks of agonizing pain in the situation of the portio dura, and extending across the cheek, and upwards to the eye, so that these parts were drawn into frightful spasms. A dry and constant cough also harassed him greatly. The paroxysms of pain were often brought on by the slightest motion of the parts, by an inconvenient attitude, by a noise, or any other trifling cause which disturbed him, and had so affected the health, that the patient was extremely emaciated, and nervous in the highest degree. Often was he afraid to eat, in case his tormentor should seize him.

This patient had suffered repeatedly from venereal affections, and M. W. was inclined to attribute his neuralgia to the virus still lurking in his system. He put him upon a mercurial course, and ordered warm baths at the same time. The friction of the unguentum hydrargyri was continued for upwards of two months, and under this treatment a complete cure was effected.

Meglin, in his "Rech. et Observ. sur la Neuralgie Faciale," Strasbourg, 1816, narrates a case of neuralgia of the portio dura, in which his pills, of extract of hyoscyamus, of wild valerian, and of oxyde of zinc, were used with excellent effects. The patient was a female, and attributed her malady to long-continued suckling.

It is important to keep in mind that, in cases of neuralgia affecting the portio dura, the muscles of the face are much more subject to a convulsive twitching or trembling, than in neuralgia of the other nerves. The explanation of this fact is beautifully in accordance with the theory of its functions, proposed by Sir Charles Bell. Paletta, the Italian surgeon, has described a form of neuralgia which is not very common. He designates it the "neuralgie mastoidienne," from the pain being confined to the mastoid process and its immediate vicinity. The following ointment, rubbed upon the part every two or three hours, generally effected a cure.

℞. Unguent. althœæ.......... ℥j.

Olei Succini.............. ʒss.

Calomel.................. ℈j. M.

Having thus selected a few cases, to illustrate the characters of this very formidable disease, as it affects the different nerves of the face, we deem it unnecessary to follow our author through its history, and the minute description of its symptoms; but shall content ourselves with a few cursory remarks. We agree with him in considering, that the only rational arrangement of the different forms of this Proteian monster is twofold, viz. into those which are regularly periodic or intermittent, and those which are not so; and, secondly, into the purely simple, nervous, or uncomplicated, and the symptomatic, or such as are connected with organic disease, either of the nerve itself, or of some other part.

Some pathologists speak of traumatic, rheumatismal, gouty, inflammatory, metastatic, gastric, carcinomatous, and syphilitic neuralgias. We have no objection to this multiplied division as only indicating the importance of always attending to the constitution and temperament of our patients in the treatment of the disease; but the same may be said of every other disease, as well as of the one under consideration. We cannot, therefore, admit each as a separate species, but rather as a shade of a species, or as a variety. How important it is, on the other hand, to ascertain the periodical and idiopathic character, or the reverse, will appear from the very different results of our treatment in the two forms: in the former, it is generally satisfactory —but, alas! in the latter, it is often quite inefficacious.

The diagnosis of neuralgia, from rheumatic, gouty, or hysterical pains, is abundantly easy. The disease is more common in cold and damp, than in warm and genial climates; and in persons between 30 and 60 years of age than at earlier periods of life. The exciting causes are very manifold, and may be either external, as a blow, wound, presence of any foreign body, tumour, or ulcer—the exposure of the face to high heats, or to chilly damp winds—the application of very cold washes, and also of acrid cosmetics, &c., or internal, among which are enumerated the metastasis of gout, or of rheumatism, the venereal virus, the sudden recession of the exanthemata, the suppression of accustomed hæmorrhages, discharges, &c. The morbid anatomy of neuralgia is, as yet, quite unsatisfactory, and perhaps it will always remain so in a large proportion of cases. Still, it is well worthy the attention of physicians to examine, by dissection, the state of the nerves, whenever they can do it. Cotugno says that he found an infiltration under the neurilema of the sciatic nerve, in one case; and other authors have told us, that the nerves which have suffered from neuralgia are red and swollen; but these appearances are by no means steady, and cannot, therefore, be admitted as the pathological character of the disease.

In the present time, we know as little of its real nature as of that of hydrophobia, or of epilepsy, or cholera, &c. The conclusion which Weisse has drawn from his experience is scientific and correct.

"Nervi autem duplici modo patiuntur: vel immediate, quando ab aliqua caussa ipsi afficiuntur; vel mediate, quando ab illa partes nervis vicinæ infestantur." And he adds: "Ad primum morborum genus prosopalgia pertinere videtur, quia præter dolorem faciei acerbum, nihil quod præternaturale habendum sit, in ulla parte deprehendimus. In ea faciei regione, quam dolor occupat, nec rubor, nec duritas, nec tumor conspicitur, et tamen dolor est vehementissimus, atque haud raro convulsiones musculorum sese adjungunt." 126.

We cannot, therefore, wonder much that the treatment of neuralgia is still often unsatisfactory, and in too many cases quite impotent; but, at the same time that we admit this, we are equally assured of the high importance of basing our "rationes medendi," as far as possible, on rigid inquiries as to the pathological state which may exist.

If symptoms of local fulness or of inflammatory action be present, (and the knowledge of this is easily ascertained by careful examination by means of pressure of the part, by the action of local warmth, and so on) the clear indication is, the abstraction of blood, and the best method by far of effecting this is by cupping; a quantity is thus speedily drained off, and an obvious impression on the vascular energies of the part is thus made at once; besides, the counter-irritation of the numerous wounds, and of the powerful suction, is of decided utility. A few brisk purges, and then a blue-pill or two, every night for a week, or two, with a dose of colchicum once or twice a day, will often succeed admirably well. When any accustomed discharge has been stopped, or any exanthematous disease been repelled, we must, as a matter of course, endeavour to recal these by appropriate treatment. In the latter case, some practitioners praise highly the decoction of the dulcamara. Sarsaparilla also is useful in such cases. We have known an emetic work a charm when other remedies had failed. The tartar-emetic ointment has been well spoken of by M. Fallot. If a syphilitic taint be suspected, a mercurial course may be necessary. A case in illustration has been reported from Masius.

When the paroxysms are distinctly and regularly periodic, cinchona or arsenic are by far the most potent means we can use. The doses must be large, often enormous before we succeed, and the use of the remedy ought to be continued for some time after the abatement or disappearance of the pain. A most useful adjunct to these medicines is some vegetable antispasmodic, as assafœtida, valerian, &c. &c. The subcarbonate of iron is well-known to be often an "heroic remedy." Hutchinson has employed it in not fewer than 200 cases of neuralgia, unattended with inflammatory symptoms, with great advantage.

As mere anodynes, hyosciamus, belladonna, stramonium, and opium, may often succeed in lulling the pain for the time; but we cannot expect to effect a radical cure with these poisons. The fetid gums, united with camphor, are sometimes very useful. Frank praises highly a combination of musk, calomel, and the golden sulphuret of antimony. The Plummer's pill may be a very good substitute. Naumann recommends strychnine in preference to all other medicines.

As to external applications, they may be useful, as affording some relief during the paroxysm; the croton and cajeput oil, strong æther, solution of camphor in spirit of turpentine, the extracts of belladonna and of stramonium have been occasionally used with advantage.

In our practice, the linimentum hydrargyri has sometimes been of decided utility; the part must be kept well anointed with it for a week or more. Electricity, galvanism, and acupuncturation have been praised by some, and condemned, as utterly useless, by others.

Magnetism of late has been again introduced as an omnipotent panacæa: we much doubt its efficacy in any severe case; still it is not fair to reject all testimony, merely because it does not tally with our notions a priori. The conclusion to which M. Thouret, more than half a century ago, arrived, is probably quite just. He says, "the effect of a magnet upon tic douloureux is feeble, although sufficiently real. It acts as a palliative, which soothes at least for the moment, the severity of the painful paroxysm. It is therefore to be viewed as a feeble remedy against a formidable disease. But amid sufferings, like those of the tic, no means of relief, however slight this may be, ought to be neglected, and as what is used one day with success may be useless on the morrow, we should thankfully receive any additition to the catalogue of our anodynes."

As to the operation of dividing the nerve, Dr. Halliday, without condemning it altogether, is not inclined to recommend it in many cases. His reasons are, that it is rare that only one branch of a nerve is affected;—that it is by no means easy to ascertain that all the fibrillæ are fairly cut through;—and that, even when they are so, re-union may take place, and the disease be thus re-established. This is not mere conjecture; but rests on numerous facts which have been published. In neuralgia of the portio dura, he thinks that it is quite inadmissible.

From the preceding analysis of this monograph, our readers will be able to judge for themselves of its merits; without any claims to originality, it presents the reader with a very correct and well-illustrated history of facial neuralgia; the symptoms, diagnosis, and treatment. Its perusal will amply repay the attentive reader, by leading him to the reflection that neuralgia, although it may always or generally exhibit the same leading phenomena, is very different in its nature in different cases, and therefore demands the greatest nicety of judgment for its appropriate treatment.

